# 

**Published:** 1888-11

**Authors:** 


### CONTENTS.

### COMMUNICATIONS.

Address of B. H. Catching.... 525

### PROCEEDINGS.

American and Southern Dental Associations.... 531

### CORRESPONDENCE.

Notes from New Zealand.... 564

### SELECTIONS.

Peculiarity of American Eyes.... 570
Distilled Water.... 570
Eye Inflammation Dependent upon Lesions of Dental Branches of the Fifth Nerve.... 571
Iodoform Paste.... 572
To Solder Steel.... 572
Mixture to Prevent Toothache.... 572
Preparation of Chalk for Dental Powders.... 573
Odontiana.... 573
Toothache—Solidified Creosote.... 573

### EDITORIAL.

A Dinner to Dr. McKellops.... 574
Ohio District Dental Societies.... 575
Dentist's Register.... 576
The Dental College.... 576

